# Fulfilling Educational Competencies through Global Pharmacy Experiences

**DOI:** 10.3390/pharmacy7020050

**Published:** 2019-05-26

**Authors:** Lindsey M. Childs-Kean, Carol Motycka, Sven A. Normann, Randell Doty

**Affiliations:** 1 Department of Pharmacotherapy and Translational Research, University of Florida College of Pharmacy, Gainesville, FL 32610, USA; lchilds-kean@cop.ufl.edu (L.M.C.-K.); normann@cop.ufl.edu (S.A.N.); 2 Department of Pharmacotherapy and Translational Research, University of Florida College of Pharmacy, Jacksonville, FL 32209, USA; motycka@cop.ufl.edu

**Keywords:** global health, international health, advanced pharmacy practice experiences

## Abstract

Many Colleges of Pharmacy in the United States offer international education and practice experiences to their students. Multiple publications have described these offerings and related them back to the CAPE 2013 Outcomes. This article describes the multiple international programs offered by one College of Pharmacy, including international Advanced Pharmacy Practice Experiences, Short Study Abroad Programs, and International Health Outreach Trips. The article also details the relevant competencies associated with these international experiences.

## 1. Introduction

Studying pharmacy education and practicing internationally has become more intriguing in Colleges of Pharmacy within the United States (U.S.). Multiple reviews and surveys have been done to capture the extent and variety of the international experiences offered to pharmacy students [1,2,3,4]. In summary, most U.S. Colleges of Pharmacy offer both didactic coursework in Global/International Health and/or at least one Advanced Pharmacy Practice Experience (APPE) at an international location. Some colleges offer a minor, certificate, or dual degree in Global Health [2].

The Global Pharmacy Education Special Interest Group of the American Association of Colleges of Pharmacy released a report tying International/Global Pharmacy Education to the CAPE 2013 Outcomes [5]. The writing group determined that the following subdomains were most relevant to International/Global Pharmacy Education: Learner, Health and Wellness, Population-based care, Problem solver, Educator, Interprofessional collaboration, Cultural Sensitivity, Communicator, Self-awareness, Leadership, and Professionalism [5]. While there has not been a publication linking the Accreditation Council for Pharmacy Education (ACPE) Standards 2016 to International/Global Pharmacy Education, many of the same domains appear in the standards as in the CAPE 2013 Outcomes [6].

The aims of this article are to describe the different international/global opportunities that students at the University of Florida (UF) College of Pharmacy have both within the Doctor of Pharmacy curriculum and through extracurricular offerings and to relate how those opportunities seek to fulfill educational competencies/standards. The opportunities within the curriculum include international APPEs, short study abroad programs, and international health outreach trips. The extracurricular offerings include the International Pharmacy Student Federation chapter, International Education Week, and Visiting Scholars.

The University of Florida is a Primary Academic Research Health Science Center with six Health Science Colleges including Dentistry, Public Health and Health Professions, Medicine, Nursing, Pharmacy, and Veterinary Medicine. The College of Pharmacy offers a 4-year post prerequisite professional Doctor of Pharmacy degree [7], as well as Master’s degrees and PhDs in five departments: Medicinal Chemistry, Pharmacotherapy and Translational Research, Pharmaceutical Outcomes and Policy, Pharmaceutics, and Pharmacodynamics. The courses outlined in this article are housed in the Department of Pharmacotherapy and Translational Research. The University supports the College’s efforts via the University of Florida International Center (UFIC), which provides logistical and administrative support for formal international courses and study programs.

## 2. International Coursework

### 2.1. International Advanced Pharmacy Practice Experiences

Advanced Pharmacy Practice Experiences (APPEs) are experiential courses offered in the final year of a College of Pharmacy curriculum. These courses emphasize continuity of care in both the community and health system settings, which will expose students to diverse patient populations as part of an interprofessional team. APPEs allow students to apply their knowledge and skills in a real-world practice setting under the supervision of a preceptor encompassing all aspects of patient care. Curricular guidelines for APPEs are provided by the Accreditation Council for Pharmacy Education (ACPE). The latest guidelines are from 2016. Standard 13 outlines the requirements and options for these experiential courses including key elements and including activities. While these guidelines require all required APPEs to be conducted in the U.S. and U.S. healthcare environments, elective APPEs can include the possibility of international experiences.

At the University of Florida College of Pharmacy, APPEs are 6 weeks in length. Students are required to complete seven rotations, totaling 1680 h. All students are required to take four specific required APPEs: Community Practice, Ambulatory Care, Hospital Practice, and General Medicine. The other three APPEs are electives. Students must complete at least two patient care electives and no more than one non-patient care elective. While an experience may be listed as a non-patient care APPE, this does not exclude students from participating in patient care. We designate all international APPEs as non-patient care so that an international experience cannot be substituted for one of the two patient care electives that are required. However, preceptors of international rotations can and do offer students patient care as part of their experiences.

In order for an international APPE site to be approved for student participation they are required to meet certain criteria, including creation of a syllabus specific to the site, nomination of a faculty preceptor for appointment to the College of Pharmacy Faculty, and a site visit by a representative of the College of Pharmacy. Each syllabus is based on a template syllabus from the UF College of Pharmacy, which covers the goals, objectives, activities, feedback, and grading criteria, as well as standardized language which covers the items required by the University and College. Here is a list of typical objective statements which might be added to the overall APPE objectives specific to international APPEs to emphasize what additional competencies a student would be expected to gain while participating in such an experience:

1. Distinguish the difference in pharmacy legal requirements, pharmacy practice, and access to and utilization of healthcare services between the US and <host country>.
2. Identify conditions and their treatments which are unique to <host country>.
3. Determine unique international pharmacy practice elements which will improve individual practice as a pharmacist in the United States.
4. Demonstrate the ability to assimilate and apply basic, clinical, and social science knowledge in the care of patients taking into consideration regional and cultural beliefs.
5. Have a better understanding of patient care in the <host country>.
6. Develop a mental framework for the patient care process as observed in the <host country>.
7. Compare and contrast patient experiences from the <host country> to U.S. pharmacy practice.
8. Communicate a personal pharmacy practice vision that incorporates elements learned from various international practices.
9. Develop and apply leadership qualities that are unique to leading a program that brings pharmacists together from other regions of the world which facilitates networking and collaboration with international pharmacy professionals.

The subdomains of the CAPE 2013 outcomes that apply to this learning experience are: Learner, Health and Wellness, Population-based care, Problem solver, Educator, Interprofessional collaboration, Cultural Sensitivity, Communicator, Self-awareness, Leadership, and Professionalism [5].

At UF, students are introduced to the possibility of international APPEs via a video that details which locations are available, the focus of the experience, housing options, etc. Students who are interested are required to submit an application and prioritization survey for review prior to APPE selection. Based on their application and their ranking of locations, students are then assigned to international APPEs until all the spaces are filled or until no students are available to assign given the needs of scheduling their other APPEs. Once assigned, students work with the UFIC to register their international travel. They also work with their preceptor and/or host site to arrange travel and housing.

Once on-site, student experiences are tailored to the site where the APPE is offered, but all of them provide insight into health care in the host country. Current international APPEs are in Germany, the United Kingdom, Spain, Finland, Australia, and Malta. Most sites offer hybrid experiences which include patient care as well as projects or research. The patient care locations typically include both inpatient and outpatient experiences.

Since 2013 UF has sent 68 students for APPEs to sites outside of the U.S., for an average of 11 student placements per year. There are currently 14 more students scheduled for international APPEs during the 2019–2020 academic year.

The number of students completing an international APPE is lower than the number of applicants. Ideally, we would like to be able to accommodate every student that applies, but this is not possible currently. Making sure the students who apply can meet all their required experiences is the first priority. Our international preceptors also have obligations to other institutions both in country and internationally that limit the number of opportunities that they can offer and the specific timing of those offerings. This then produces a limited window to place the students where their schedule and the schedule of the preceptors match.

International APPEs are the longest and most in-depth experience that we are able to provide as part of the professional degree program. Not every student is capable of taking advantage of this opportunity, even if we had additional opportunities available to them. Based on informal student feedback, there are several barriers that a student must overcome in order to participate. The most prominent is cost. Even though many of our sites provide low cost housing alternatives, the expenses for six weeks of daily living and the travel costs are enough to limit these experiences to a small group of students. Another barrier is flexibility. In order to be able to complete an international APPE, a student has to be able to leave the country for six weeks. Their lives must allow for such flexibility to off load any responsibilities they have at home. Things such as childcare, work, pets, and family responsibilities all need to be arranged; otherwise, participation will be unlikely. In a typical year, only about 20 students are able to apply for the international APPEs because most cannot find a clear way to do so. Therefore, while we will continue to expand the possibilities for international APPEs, there will always be a limit to the number of students who have the ability to participate. This then limits the number of students who can gain the advantage that this program offers to them in terms of international competencies. Therefore, additional mechanisms must be found if we want to expand the opportunities to learn about how healthcare works in other countries and cultures.

### 2.2. Short Study Abroad Programs

The college wanted to provide pharmacy students an opportunity to see the practice of pharmacy in other countries around the world without requiring completion of a six week international APPE. In order to do this, we offer “Short Study Abroad” programs (SSAPs). These programs allow students a glimpse into the healthcare system in other countries, which provides a perspective for their own practice of pharmacy when they return to the United States. Originally, the programs began as three-week trips, but were later shortened to two weeks, which made travel for the faculty leaders involved easier. The trips take place after the students complete all of their coursework for the year (typically in May), and any student in good academic standing may participate in the program if they are chosen following an application which includes an essay describing why they would like to participate in the program. Students rank which trip they would like to participate in and faculty involved in assigning the trips do their best to place them in either their first or second ranked choice. Each program involves 8–12 students and one faculty member. In a typical year, three to four programs are offered. As of this writing, students have travelled to Italy, Malta, the United Kingdom, Ireland, Scandinavia (Denmark, Finland, Norway, and Sweden), Thailand, China, Japan, Australia, Germany, and the Philippines.

All SSAPs operate under the same syllabus as part of an elective course. Students can take this elective more than once, but only the first such course is allowed to be credited against the required number of elective courses for their Pharm.D. degree. Like the APPE syllabus, the SSAP syllabus is based on a template syllabus. Learning objectives, competency, and assessment criteria are included in the SSAP elective course syllabus:

1. Communicate with pharmacy students and faculty, and other members of inter-professional healthcare teams (when possible) to acquire knowledge about differences in culture and professional customs and issues concerning public health problems and policy, and the medication use system.
2. Demonstrate cultural competency with pharmacists and other healthcare providers encountered in the international setting.
3. Compare and contrast pharmacy education in the United States with pharmacy education in the program country.
4. Compare and contrast the role of a pharmacist in a variety of practice settings (such as community, health-system, ambulatory care, and industry) in the United States with the role of a pharmacist in those settings in the program country.
5. Recognize cross-cultural differences that impact a patient’s beliefs about health and treatment of illnesses.
6. Demonstrate the professional attitudes and behaviors that characterize a professional pharmacist.
7. Demonstrate the personal development expected of a pharmacy student by showing initiative, confidence and adaptability.

The subdomains of the CAPE 2013 outcomes that apply to this learning experience are: Learner, Health and Wellness, Population-based care, Problem solver, Educator, Interprofessional collaboration, Cultural sensitivity, Communicator, Self-awareness, Leadership, and Professionalism [5].

There are three components of the course: Pre Program, In Country Program, and Post Program. Each has tasks that the students need to perform.

#### 2.2.1. Pre Program Activities

Prior to traveling to the countries the team will be visiting, the students are responsible for preparatory work. Some of this work is universal to the course, and some is specific to the particular program. Course content is managed via an online course management system as well as program-specific private Facebook groups.

All students must participate in an orientation session. Materials at this orientation cover information mandated by the UFIC regarding safety, security, and protocols for a UF student studying abroad. Faculty also include information to make the program run smoothly including emphasizing responsibility, timeliness, courtesy, safety, and communication.

All students must complete readings specific to their program, which cover both topics that are overarching regarding health care in the country they are travelling to or specific about some notable problem that they have had to deal with in recent history. These readings are solicited from our hosts for each program. Students must complete a quiz over this material prior to leaving for the in country program to ensure they have read and understood the material.

Students are also required to form into groups of 2–3 students and prepare a short 10–15 min presentation. The audience for this presentation will be the host faculty, students, pharmacists, etc. that they will meet while in country. The topics cover aspects of practice and education in the United States that might be of interest to the hosts. The following list of possible topics is provided to the students, but they can also choose other topics upon approval of the faculty supervisor.

- Why you chose pharmacy
- What you are studying/learning in pharmacy school (1 PD/2 PD curriculum)
- What pharmacists do in the U.S.-scope of practice (immunizations, MTM, etc.)
- Different career paths in pharmacy in the U.S.
- What makes you proud/excited to be a pharmacy student and a Gator
- Legislative advocacy by pharmacy students at UF COP
- Pharmacy student organizations and community service at the UF College of Pharmacy
- Current Topics in Pharmacy Practice in the U.S.

Examples:

○ The current status of pharmacists as providers in the U.S.
○ Current state of opiate control and prescribing patterns in Florida

Students are expected to have these presentations ready before leaving the U.S. and to have practiced them. They are instructed to be ready to present them at any point and possibly multiple times during their two weeks in country.

#### 2.2.2. In Country Activities

While in country, students experience different aspects of pharmacy, ranging from patient care to the pharmacy education process. Some examples of what a student may experience include: visiting a hospital pharmacy and meeting with the pharmacists to tour and discuss their practice and drug distribution system; visiting a community pharmacy to discuss what medications may be provided to patients with or without a prescription and discuss the daily pharmacy operations; visiting a local university to tour and spend time in a classroom followed by discussion of life as a pharmacy student in their country subsequently followed by student presentations often by both the visiting students and the hosts; visiting the local professional pharmacy organization and discussing their role within their country; and visiting the local equivalent of the U.S. Food and Drug Administration for their country (e.g., Italian Medicines Agency: AIFA).

Although the programs are geared towards learning about the other country’s profession of pharmacy, students are also encouraged to explore the culture of the country as well to get a better understanding of the people within those countries and the way they live every day. This information helps them in turn better understand why their healthcare system may be set up the way that it is. Students experience the local cuisine, local cultural experiences, and of course the local historic sites. Some of these have included a visit to the leaning Tower of Pisa, a boat ride on the River Thames, a trip through the Highlands in Scotland, a festival in Norway for Constitution Day, and even a visit to the Hofbrauhaus. Including these important cultural experiences adds to the overall understanding of the country and provides the students a glimpse of what life may be like outside the U.S.

While in country, the students are required to keep a daily journal of their reflections regarding their experiences for the day, both healthcare-related and cultural. These journals are submitted as an assignment for their post program work.

#### 2.2.3. Post Program Activities

Once the in country portion of the program is over, there are two assignments that are required to be turned in within 20 days of the end of the in country program. One is the digital journal, which was mentioned previously, and the other is a video assignment. The video assignment consists of a video that each student produces. Drawing upon the student’s own reflections from their digital journal, each student records a video summary of their experiences during the in country portion of the course. The video is required to be 20–30 min in length. Students are instructed to use their trip experiences and apply them to one of the following scenarios:

- Imagine you are preparing to speak to your government representative (senator or house of representatives delegate), choose one issue in pharmacy practice and provide possible solutions based on what you have learned during the trip.
- You are presenting to a patient who is an immigrant from one of the places you have just visited. Explain how to navigate the U.S. healthcare system compared to theirs.
- One of your patients’ companies is moving them to the country you just visited for 6 months. They know you just visited there and want your help in understanding what they should expect when using the healthcare system in that country.
- Upon your return you have been asked to present to your local chapter of IPSF regarding the differences that you saw between the U.S. healthcare system and the healthcare system of the country you visited.

Alternatively, a student may choose to focus on some other specific subject of interest to them which sparked their interest during the program, subject to faculty leader approval.

Students’ digital journals and video summaries are evaluated by the faculty supervisor of their specific program using a rubric. Utilizing their pre program quiz score and their in country evaluation by their faculty, this then creates a grade for the student for the course. This ultimately determines their pass/fail status for the course.

Students who have participated in these SSAPs described the experience as “life changing”. Overall, the informal feedback has been extraordinary, and some students feel it may be the greatest experience they have throughout pharmacy school. By allowing them a chance to see healthcare in an entirely different light, we are providing them an opportunity to take pieces of what they learn and bring them back to the U.S. to in turn help improve our overall healthcare system.

### 2.3. International Health Outreach Trips

For several years, students, faculty, and members of the community have conducted medical mission trips to Mexico, Central America, and South America. These student-led trips primarily involved the College of Medicine with some participation by students in the Colleges of Pharmacy and Dentistry. Over the years, the trips expanded to include Audiology, Nursing, and Physical Therapy. In recent years, students on the College’s Orlando campus have joined medical, nursing, and social work students from the University of Central Florida on similar trips to the Dominican Republic and Peru. Since 2013, over 300 pharmacy students have participated in these trips, averaging about 45 students per year. The goal of these trips is to provide care to underserved populations in cooperation with local educational and/or service-related organizations. These week-long trips take place during a mid-term break in the Spring semester. The various trips are largely funded through solicited donations by students coming primarily from family and friends.

Students are selected through an application process, and student coordinators and faculty are involved in the final selection of team members. Students must be in good academic standing to participate. Most participating students are in their second or third year of the pharmacy program; however, interested first and fourth year students are occasionally able to participate.

Once selected, students are enrolled in a two-credit elective course, half of which consists of online readings, videos, conference calls involving live discussion, and reflection papers. The other half of the course is related to the in-country experience. Learning objectives, competency and assessment criteria are included in a course syllabus:

1. Provide patient-centered care in cooperation with other health professional health care team members based upon sound therapeutic principles and evidence-based data, taking into account relevant, social, cultural, and economic issues.
2. Describe the pathophysiology and characteristics of typical infectious diseases, common acute and chronic illnesses, common typical injuries and/or common nutritional problems that are likely encountered in the assigned outreach experience.
3. Demonstrate cultural competency with patients and local healthcare providers in the international setting.
4. Recognize cross-cultural differences that impact a patient’s beliefs about health and treatment of illnesses.
5. Demonstrate the professional attitudes and behaviors that characterize a professional pharmacist.

The subdomains of the CAPE 2013 outcomes that apply to this learning experience are: Learner, Health and wellness, Population-based care, Problem solver, Educator, Interprofessional collaboration, Cultural sensitivity, Communicator, Self-awareness, Leadership, and Professionalism [5].

The role of pharmacy participants before the trip is primarily to provide medications used on the trip. Students gain experience developing a formulary for the trip’s healthcare services. The formulary primarily includes oral and topical medications. For chronic conditions, it is the philosophy of traveling teams to not dispense medications that are not available in the host country. It is also the philosophy, whenever possible, to follow standards of care and practice that exist in the U.S. [8]. Participating students raise funds for the purchase of the medications on the formulary. Medications include both prescription and non-prescription items. The most common items are multivitamins for children and adults and analgesics, such as acetaminophen and ibuprofen. While most items are purchased from organizations that provide medicines and medical equipment for medical missions, some are donated by local pharmacies and pharmaceutical manufacturers.

While in-country, most of the experiences involve teams providing acute care ranging from treating minor injuries and infections (including parasites) to minor medical procedures. A pharmacist preceptor supervises the student(s) at all times. All patients are interviewed by the student and counseling is provided on each medication provided. A brief history is obtained, including allergies, etc. If the student or provider does not speak Spanish, a translator is utilized. While some students are bilingual, local translators are often utilized due to regional dialects. While some prescription medications are acquired in the U.S., most are purchased in the host country upon arrival or through local partners. No controlled substances are dispensed. Medications that are not used are donated to local partners.

After completion of the trip, students participate in multiple discussion and reflection sessions. Topics for these sessions include patient care or practice pearls and examples of unexpected learning.

For many students, this is their first international travel experience. During these experiences, students are exposed to culture and language, interprofessional practice, healthcare ethics, and first-hand exposure to social determinants of health, including the impact of economics, food, education, environment, health care. Many students have described these experiences as life-changing.

## 3. Local Initiatives

As mentioned above, these programs, while extensive, are limited in the numbers that can participate, both by the number of opportunities available but also by any given student’s ability to have the money and flexibility to participate. So where does that leave all those students who will not have such opportunities? We feel strongly that providing alternative experiences locally is one thing we can do to make up the gap in understanding between those students that can go abroad and those that cannot. We have, therefore, taken steps to provide opportunities for students to participate locally in events which will at least expose them to different ways of thinking, different modes of pharmacy practice, and the different advantages and barriers that are existent in other healthcare systems. We will outline some of the ways that this is currently being accomplished.

### 3.1. International Pharmaceutical Student Federation (IPSF) Involvement

At UF, IPSF is folded into the American Pharmacists Association-Academy of Student Pharmacists (APhA-ASP). Our APhA-ASP chapter has a vice president for IPSF who coordinates IPSF activities. Our IPSF chapter independently organizes seminars for students to learn from students, faculty, and other practitioners from foreign countries whenever these opportunities arise. In recent years, a young pharmacist from Argentina and the IPSF President from Germany conducted such seminars. They are also pivotal in organizing such seminars in conjunction with the other programs listed below. The IPSF chapter also provides support for local Migrant Farmworker Health Fairs each Spring. UF students have also attended international conferences, such as the IPSF World Congress, the IPSF PanAmerican Regional Symposium, and the International Pharmacy Federation (FIP) World Congress, dating back several years.

### 3.2. International Education Week

UF participates in International Education week each November. The College of Pharmacy has participated in this event since 2015. Each year we bring in a prominent educator/practitioner to our college to present two seminars and to participate in classes, discussions regarding research, and teaching collaborations, as well as social events. During the week, the guest presents a seminar specifically directed at students to help them better understand healthcare in their home country. All students are invited to attend, and the seminar is broadcast live to our distance campuses at a time when any student could attend.

### 3.3. Visiting Students and Scholars

During any given year, we have exchange students or other short-term visiting students as well as visiting graduate students. When possible, we set up seminars for these students to talk to our students about the practice of pharmacy in their home country. Like the seminars for International Education Week, these seminars are also broadcast to all campuses. We regularly have visiting students from Spain, Thailand, Japan, and Germany who deliver such seminars.

### 3.4. Visiting Student Participation in Academics

When visiting students come to UF for APPEs, we regularly give them the opportunity to participate as facilitators for coursework. This allows our students to have in-class contact with students from a different culture and a different practice background. Likewise, we also have students in exchange programs that come to our college for the entire first year of their curriculum. They are completely involved with our first-year students in every aspect of the curriculum both in class and through extra-curricular activities.

So, while we cannot provide every student with the opportunity to learn about healthcare outside of the U.S. via first hand observation of that healthcare system, we can and do provide the opportunity for any interested student to learn about such topics from the people who experience it as native users.

## 4. Discussion

International educational experiences are valuable for pharmacy students in many different ways. As shown in this article, such international culture and healthcare exposure provides unique opportunities to enhance the attainment of curricular objectives and specific competencies in pharmacy students. The three current formalized international programs allow many UF College of Pharmacy students to have these experiences first hand and thereby meet the additional learning objectives that can be acquired via international opportunities. It is also important to structure as many experiences as possible for those students who cannot participate in a formal international program first hand so that they too have the opportunity to grow toward the same objectives and competencies. Future directions for the international programs at UF College of Pharmacy include a formal assessment of learning outcomes using former student-provided feedback, continuous overall quality improvement reviews of the current programs, and expansion of the offerings we currently provide to introduce new countries and new experiences to our students. We hope that through our continuing international efforts, we may be able to improve not only the overall health care education that our students receive, but in turn provide a bit of education to others around the globe as well.
